# Incidence, timing and outcome of AKI in critically ill patients varies with the definition used and the addition of urine output criteria

**DOI:** 10.1186/s12882-017-0487-8

**Published:** 2017-02-20

**Authors:** J. Koeze, F. Keus, W. Dieperink, I. C. C. van der Horst, J. G. Zijlstra, M. van Meurs

**Affiliations:** Department of Critical Care, University of Groningen, University Medical Center Groningen, Postbus 30.001, 9700 RB Groningen, The Netherlands

**Keywords:** Acute kidney injury, Definitions, Incidence, Intensive Care, Timing

## Abstract

**Background:**

Acute kidney injury (AKI) is a serious complication of critical illness with both attributed morbidity and mortality at short-term and long-term. The incidence of AKI reported in critically ill patients varies substantially with the population evaluated and the definitions used. We aimed to assess which of the AKI definitions (RIFLE, AKIN or KDIGO) with or without urine output criteria recognizes AKI most frequently and quickest. Additionally, we conducted a review on the comparison of incidence proportions of varying AKI definitions in populations of critically ill patients.

**Methods:**

We included all patients with index admissions to our intensive care unit (ICU) from January 1^st^, 2014 until June 11^th^, 2014 to determine the incidence and onset of AKI by RIFLE, AKIN and KDIGO during the first 7 days of ICU admission. We conducted a sensitive search using PubMed evaluating the comparison of RIFLE, AKIN and KDIGO in critically ill patients

**Results:**

AKI incidence proportions were 15, 21 and 20% respectively using serum creatinine criteria of RIFLE, AKIN and KDIGO. Adding urine output criteria increased AKI incidence proportions to 35, 38 and 38% using RIFLE, AKIN and KDIGO definitions. Urine output criteria detected AKI in patients without AKI at ICU admission in a median of 13 h (IQR 7–22 h; using RIFLE definition) after admission compared to a median of 24 h using serum creatinine criteria (IQR24-48 h). In the literature a large heterogeneity exists in patients included, AKI definition used, reference or baseline serum creatinine used, and whether urine output in the staging of AKI is used.

**Conclusion:**

AKIN and KDIGO criteria detect more patients with AKI compared to RIFLE criteria. Addition of urine output criteria detect patients with AKI 11 h earlier than serum creatinine criteria and may double AKI incidences in critically ill patients. This could explain the large heterogeneity observed in literature.

**Electronic supplementary material:**

The online version of this article (doi:10.1186/s12882-017-0487-8) contains supplementary material, which is available to authorized users.

## Background

Acute kidney injury (AKI) is a serious complication of critical illness with both attributed morbidity and mortality at short and long-term [1–7]. The incidence of AKI reported in critically ill patients varies substantially [4, 8]. Before 2004 the diagnosis of AKI was based on urine output and markers of decreased urinary waste secretion i.e., serum urea and creatinine as markers of a decreased glomerular filtration rate (GFR), but differed per study. In 2004 an attempt was made to standardize outcomes of studies by unifying the definition of AKI using the Risk, Injury, Failure, Loss and End-stage (RIFLE) definitions [9]. In 2007 adjustments resulted in the Acute Kidney Injury Network (AKIN) definition, driven by observations that minor increases in serum creatinine over a shorter time period are also associated with adverse effects. The current definition made by Kidney Disease Improving Global Outcomes (KDIGO) is similar to the AKIN definition but the time frame is extended from 48 h to 7 days [10, 11]. Serum creatinine is considered an inferior marker of kidney function during critical illness, as the rise in serum creatinine is commonly delayed after kidney function decline [12, 13]. Adding urine output criteria to creatinine levels increases AKI incidences compared to criteria using serum creatinine levels only [14–18]. However, many studies dismiss hourly urine output data as this data is often hampered by frequent missing values. Consequently, studies may report AKI using three different definitions, all with or without urine output criteria. Comparison of published studies is hampered by this dissimilarity.

### Objective

We aimed to assess the difference in AKI incidence using the three definitions, with and without urine output criteria, and to assess which AKI definition recognizes AKI first. Second, we aimed to assess differences in patient characteristics in patients with AKI according to the three definitions. Additionally, we conducted a review on incidence proportions of AKI varying with definitions in critically ill patients.

## Methods

### I Cohort study

We included all patients with index admissions to our intensive care unit (ICU) from January 1^st^, 2014 until June 11^th^, 2014. Informed consent was waived by the institutional Review Board of our hospital because the study had an observational design and all data were de-identified (METc University Medical Center Groningen, Medical Ethical Committee, chairman Prof. dr. W. A. Kamps,reference number 2013–174).

#### Data collection

Baseline data were recorded for all patients, including age, sex, Body Mass Index (BMI), admission category and type (medical and surgical; scheduled and emergency), the presence of diabetes mellitus, and chronic kidney disease. We recorded mortality and length of stay (ICU and hospital). Serum creatinine was measured each day and urinary output was recorded hourly. Reference creatinine was based on the calculated ideal serum creatinine assuming a clearance of 75 ml/min/1.73 m2 using the Modification of Diet in Renal Disease (MDRD) formula. Missing hourly urine output data was imputed based on averages using the first known value over the missing hours. AKI timing and incidence was estimated up to the first seven ICU admission days.

#### Definitions of AKI

AKI during the first 7 days of ICU admission was defined according to the RIFLE, AKIN and KDIGO definitions. Using serum creatinine with and without urine output criteria. The definitions are described in Additional file 1: Table S1.

#### Comparison of AKI definitions

RIFLE uses Glomerular Filtration Rate (GFR) criteria in addition to serum creatinine and urine output criteria. GFR criteria were abandoned in the AKIN and KDIGO definitions. The Risk, Injury and Failure categories of the RIFLE definition were replaced by AKIN and KDIGO stages 1, 2 and 3. In AKIN stage 1 an absolute rise in serum creatinine of more than 26.4 μmol/l was added to the relative increase of 150–200% in serum creatinine compared to baseline. This increase in serum creatinine of more than 26.4 μmol/l in AKIN stage 1 was replaced by an absolute rise in serum creatinine of more than 26.5 μmol/l in stage 1 of the KDIGO definition. Both in the RIFLE and KDIGO definitions the increase in creatinine is defined to occur within 7 days, which contrasts with the 48 h used in the AKIN definition.

In stage 3 of the AKIN and KDIGO definitions the need for renal replacement therapy (RRT) was added. The categories Loss and End Stage Kidney disease or equivalent (outcome) categories from the RIFLE definition were removed from the AKIN and KDIGO definitions. Urine output criteria are similar in RIFLE, AKIN and KDIGO definitions.

#### Statistical analyses

Incidence proportions of AKI were based on definitions of AKI criteria during the first week of ICU admission. Baseline characteristics were reported by proportions and means (with standard deviations) or medians (with inter quartile ranges) according to normality or skewed distributions. Normality was tested using the Kolmogorov-Smirnov test. Data were statistically tested using students t-test, MannWhitney U test or Chi square test when appropriate. In case of differences in incidences between the three AKI definitions a MANOVA post hoc analysis with the Least Significance Difference (LSD) of patient groups was performed. SPSS (IBM 2013, version 22) was used.

### II Review

Search

We conducted a sensitive search in PubMed concerning the comparison of RIFLE, AKIN and KDIGO in critically ill patients (see Additional file 1 for search terms). Only studies with adult patients that compared incidence proportions using two or more AKI definitions were considered.

## Results

### I Cohort study

#### Patients

**Fig. 1:**
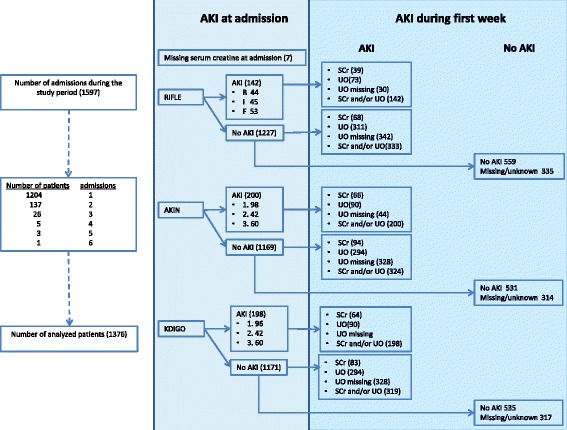
Flowchart with numbers of patients included in the study with AKI at admission and AKI during the first week of admission according to the RIFLE, AKIN and KDIGO definitions. *Left column*: patients screened for inclusion. *Middle column*: patients with AKI at admission according to the three definitions and severity. *Right column*: numbers of patients with AKI during the first week of ICU admission according to the three definitions, severity and with or without urine output criteria. Grouped by patients with AKI at admission and patients without AKI at admission; RIFLE: ‘risk’, ‘injury’, ‘failure’, ‘loss’ and ‘end-stage’ definition. AKIN: acute kidney injury network definition. KDIGO: kidney disease improving global outcome definition. SCr = serum creatinine, UO = urine output

The study was performed in a 42-bed critical care department in a tertiary referral hospital with 3561 admissions in 2014. In the study period 1376 patients were admitted with a total of 5734 observation days. 172 patients were readmitted once or more during the study period. A total of 221 readmissions were excluded for further analysis (Fig. 1).

The mean age was 60 years and 63% of patients were male. A surgical intervention preceded admission in 66% of the patients of which 80% were planned. A total of 221 patients (16%) had diabetes and 58 patients (4.2%) had chronic kidney disease (CKD) including 24 patients (1.7%) on chronic renal replacement therapy (RRT). ICU mortality was 5.6% and the median length of ICU stay was 2 days (IQR 2–3) (Table 1).

**Table 1:** Basic patient characteristics

Characteristic of patients (*N* = 1376)	
Age (years; mean (sd))	60 (16)
Male sex (N (%))	864 (62.8)
Body Mass Index (kg/m2, mean(sd))	26 (5.2)
APACHE IV (mean(sd))	52 (25)
Admission type	
• Medical (N (%))	461 (33.5)
• Surgical (N (%))	914 (66.4)
◦ scheduled (N (%))	727 (52.8)
◦ emergency (N (%))	187 (13.6)
Co morbidity	
• diabetes mellitus (N (%))	221 (16.1)
• chronic kidney disease (N (%))	58 (4.2)
• chronic dialysis (N (%))	24 (1.7)
ICU LoS (days; median [IQR])	2 [2.0–3.0]
ICU mortality (N (%))	77 (5.6)
Hospital mortality (N (%))	129 (9.4)
Serum creatinine at admission (μmol/l; median [IQR]) (*N* = 1369)	73 [58–93]
Acute kidney injury in first 7 days of admission	
• RIFLE (N (%))	210 (15.2)
◦ With UO criteria (N (%)) • AKIN (N (%))	475 (34.5) 294 (21.4)
◦ With UO criteria (N (%))	524 (38.1)
• KDIGO (N (%))	281 (20.4)
◦ With UO criteria (N (%))	517 (37.6)
• Urine output RIFLE (N (%))	265 (19.3)
• Urine output AKIN (N (%))	230 (16.7)
• Urine output KDIGO (N (%))	236 (17.2)
Renal replacement therapy (N (%))	62 (4.5)

*IQR* interquartile range

*N* number of patients

*APACHE IV* Acute Physiology and Chronic Health Evaluation IV

*ICU* intensive care unit

*LoS* length of stay

*UO* urine output

#### Total prevalence of AKI varies with the definition

A total of 107 patients (7.8%) met the criteria for AKI using the RIFLE definition based on serum creatinine and 384 patients (28%) met the criteria based on urine output during the first week of ICU admission. The combination of serum creatinine and urine output using the RIFLE definition resulted in 475 patients (35%) with AKI. For AKIN 160 patients (12%) met the criteria for AKI based on serum creatinine and 384 patients (28%) met the criteria based on urine output during the first week of ICU admission. The combination of serum creatinine and urine output using the AKIN definition resulted in 524 patients (38%) with AKI. Using the KDIGO definition 147 patients (11%) met the criteria for AKI based on serum creatinine and 384 patients (28%) met the criteria based on urine output during the first week of admission. The combination of serum creatinine and urine output using the KDIGO definition resulted in 517 patients (38%) with AKI (Table 1, Fig. 1 and Additional file 1: Table S2).

The patients only fulfilling AKI definitions based on urine output criteria more frequently had diabetes mellitus and were predominantly admitted after scheduled surgery compared to patients who fulfilled AKI criteria based on their serum creatinine (Table 2).

**Table 2:** Patient characteristics and outcome of patients grouped by AKI definition and on positive urine output criteria alone or with serum creatinine criteria

Definition	RIFLE (*N* = 326)			AKIN (*N* = 310)			KDIGO (*N* = 308)		
	UO only (*N* = 265)	UO and SCr (*N* = 61)	*p*-value	UO only (*N* = 230)	UO and SCr (*N* = 80)	*p*-value	UO only (*N* = 236)	UO and SCr (*N* = 72)	*p*-value
Characteristics of patients									
Age (years; mean (sd))	62 (15)	63 (15)	0.753	62 (15)	62 (15)	0.978	62 (15)	63 (14)	0.478
Male sex (N (%))	173 (54)	34 (10)	0.185	149 (48)	45 (15)	0.182	153 (50)	41 (13)	0.265
Body Mass Index (kg/m2; mean (sd))	27 (5)	26 (6)	0.184	27 (5.2)	26 (5.7)	0.051	27 (5.3)	26 (5.8)	0.118
APACHE IV score (mean (sd))	54 (25)	75 (22)	0.000	52 (23)	72 (26)	0.000	52 (23)	74 (26)	0.000
Admission type									
• medical (N (%))	105 (32)	22 (6.7)	0.009	88 (28)	31 (10)	0.052	95 (31)	22 (7.1)	0.006
• surgical									
◦ scheduled (N (%))	124 (38)	21 (6.4)		112 (36)	30 (9.7)		112 (36)	30 (9.7)	
◦ emergency (N (%))	36 (11)	18 (5.5)		30 (9.7)	19 (6.1)		29 (9.4)	20 (6.5)	
Co morbidity									
• diabetes mellitus (N (%))	47 (14)	11 (3.4)	1	40 (13)	12 (3.9)	0.729	41 (13)	11 (3.6)	0.857
• chronic kidney disease (N (%))	3 (0.9)	2 (0.6)	0.236	2 (0.6)	3 (0.9)	0.110	2 (0.6)	3	0.085
• chronic dialysis (N (%))	0 (0)	0 (0)	na	0 (0)	0 (0)	na	0 (0)	(0.97) 0 (0)	na
ICU Length of stay (days; median [IQR])	3 [2–6]	6 [3–11]	0.000	3 [2–5]	6 [3–11]	0.000	3 [2–6]	5 [3–9.8]	0.000
ICU mortality (N (%))	13 (4.0)	6 (1.8)	0.139	8 (2.6)	11 (3.5)	0.002	10 (3.2)	9 (2.9)	0.021
Hospital mortality (N (%))	24 (7.4)	13 (4.0)	0.012	17 (5.5)	19 (6.1)	0.000	19 (6.2)	17 (5.5)	0.001

*RIFLE* ‘risk’, ‘injury’, ‘failure’, ‘loss’ and ‘end-stage’ definition

*AKIN* acute kidney injury network definition

*KDIGO* kidney disease improving global outcome definition

*UO* urine output. SCr: serum creatinine

*N* number of patients

*P-*value difference between patients with only urine output criteria positive and patients with both criteria positive

Eighty four patients were detected on admission as having AKI using the serum creatinine criteria of the AKIN and KDIGO definitions who remained undetected using the RIFLE definition. Fourteen patients were recognized as having AKI only when using the AKIN definition (Table 3). One patient was detected as having AKI only with the KDIGO definition. (not included in Table 3). These patients differed in age, severity of illness, admission type, comorbidity, ICU length of stay and mortality. Patients only detected by AKIN and KDIGO were less ill and were admitted more frequently after scheduled surgery compared to the other groups (Table 3).

**Table 3:** Patient characteristics and outcome of patients grouped by the definition used to classify AKI by serum creatinine criteria

Characteristic of patients	Group 1 No AKI *N* = 1078	Group 2 RIFLE, AKIN & KDIGO AKI *N* = 212	Group 3 AKIN & KDIGO AKI *N* = 70	Group 4 AKIN AKI *N* = 14	*p* value
Age (years; mean (sd))	63 (19)	67 (20)	68 (15)	54 (28)	<0.05 (*)
Male sex (N (%))	681 (63.1)	122 (57.5)	52 (74.3)	8 (57.1)	0.078
Body Mass Index (kg/m2; mean (sd))	25.8 (5.6)	26.2 (5.6)	26 (6.2)	25.6 (4.7)	0.013 (****)
APACHE IV score (mean (sd))	43 (25)	76 (30)	56 (26.5)	51.5 (36.5)	0.001 (***)
Admission type					
• Medical (N (%))	308 (28.6)	114 (53.8)	26 (37.1)	12 (85.7)	0.001
• Surgical					
◦ Scheduled (N (%))	640 (59.4)	53 (25)	32 (45.7)	2 (14.3)	
◦ Emergency (N (%))	129 (12)	45 (21.2)	12 (17.1)	0 (0)	
Co morbidity					
• diabetes mellitus (N (%))	149 (13.8)	62 (29.2)	9 (12.9)	1 (7.1)	0.001
• chronic kidney disease (N (%))	5 (0.5)	48 (22.6)	4 (5.7)	0 (0)	0.001
• chronic dialysis (N (%))	0 (0)	23 (10.8)	1 (1.4)	0 (0)	0.001
ICU Length of stay (days; median [IQR])	2 (2–3)	4 (2–8)	2 (2–4)	13 (4–13)	0.001 (****)
ICU mortality (N (%))	37 (3.4)	30 (14.1)	7 (10)	3 (21.4)	0.001
Hospital mortality (N (%))	64 (5.9)	51 (24.1)	11 (15.7)	3 (21.4)	0.001

Group 1: patients without AKI

Group 2: patients with AKI according to RIFLE, AKIN and KDIGO criteria

Group 3: patients with AKI according to AKIN and KDIGO

Group 4: patients with AKI according to AKIN

*IQR* interquartile range

*N* number of patients

*APACHE IV* Acute Physiology and Chronic Health Evaluation IV

*ICU* intensive care unit

*LoS* length of stay

(*) group 1 vs group 2 and 3, group 2 vs group 1 and 4, group 3 vs group 1 and 4

(***) group 2 vs group 1, 3 en 4, group 3 vs group 1 & 2

(****) group 1 vs group 2 and 4, group 3 vs group 1 and 2, group 4 vs group 1, 2 and 3

#### Incidence of AKI varies with the definition

Of all patients who had AKI during the first week of ICU admission 142 patients (10%) were admitted with AKI according to the RIFLE definition, 200 patients (15%) according to the AKIN definition and 198 patients (14%) according to the KDIGO definition (Fig. 1). Of all patients without AKI according to the RIFLE definition at ICU entry 68 patients (4.9%) developed AKI based on RIFLE serum creatinine criteria. When the AKIN definition was used 94 patients (6.8%) developed AKI and when the KDIGO definition was used 83 patients (6.0%) developed AKI (Fig. 1 and Additional file 1: Table S2).

The severity of AKI during the first week of ICU admission varied with the definitions used.

RIFLE stage ‘risk’ occurred in 67 patients (4.9%) using the creatinine criteria solely and in 230 patients (17%) when combined with the urine output criteria. For RIFLE stage ‘injury’ this was 62 patients (4.5%) and 144 patients (10%), respectively, and for RIFLE stage ‘failure’ 81 patients (5.9%) and 86 patients (6.3%), respectively.

Incidence of AKIN stage 1 occurred in 137 patients (10%) based on the creatinine criteria and in 272 patients (20%) when combined with the urine output criteria. For AKIN stage 2 this was 60 patients (4.4%) and 126 patients (9.2%), respectively, and for AKIN stage 3 this was 97 patients (7.0%) and 108 patients (7.9%), respectively.

KDIGO stage 1 occurred in 127 patients (9.2%) based on the creatinine criteria and in 271 patients (20%) when combined with the urine output criteria. For KDIGO stage 2 this was 50 patients (3.6%) and 119 patients (8.6%), respectively, and for KDIGO stage 3 this was 104 patients (7.6%) and 106 patients (7.7%) respectively (Additional file 1: Table S2).

#### Timing of detection of AKI varying with definitions

**Fig. 2:**
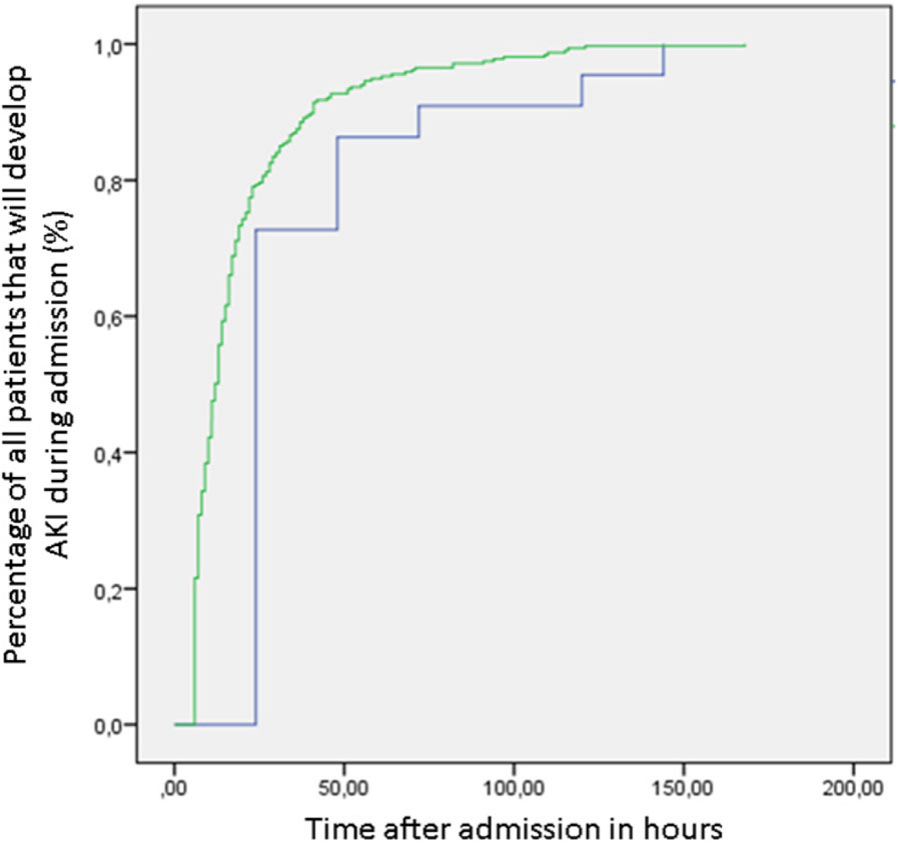
Proportions of patients who develop AKI according to RIFLE definition during ICU admission. Proportions of patients who develop AKI according to RIFLE definitions based on urine output criteria (green line) and serum creatinine criteria (blue line) plotted against the time in hours since admission to the ICU. Only patients without AKI at ICU admission who developed AKI within the first week of ICU admission are included in this graph

For urine output criteria, the median time until detection was 13 h with all three definitions (RIFLE IQR 7–22 h, AKIN IQR 7–23 h, KDIGO IQR 7–23 h). Timing and incidence of AKI based on serum creatinine criteria is comparable between RIFLE, AKIN and KDIGO in the first 48 h of admission (Additional file 1: Figure S1). AKI was detected after a median of 24 h (IQR 24–48) using the serum creatinine criteria for RIFLE, AKIN or KDIGO definitions (Fig. 2).

### II Review

A total of 383 hits were screened and 24 papers that compared RIFLE, AKIN and/or KDIGO definitions were selected for inclusion (Additional file 1: Table S3). One study was published as abstract only [19]. Fourteen studies evaluated incidences of AKI in critically ill patients and found proportions varying from 34% to 81% using RIFLE definition, from 29% to 77% using AKIN definition and from 38% to 51% using KDIGO definition.

#### AKI incidences in critically ill patients in prospective studies

Six studies used a prospective design to evaluate AKI incidences based on two or more definitions. Two studies compared all three AKI definitions RIFLE, AKIN and KDIGO and two other studies compared two AKI definitions. All studies excluded specific subgroups from their studies when defining AKI incidences. Three studies used urine output criteria in addition to creatinine for defining AKI.

Luo et al., evaluated AKI based on RIFLE, AKIN and KDIGO in 3107 patients. This study excluded patients who stayed in the ICU for less than 24 h and patients with end stage kidney disease, patients on RRT and patients who had a renal transplant in the preceding 3 months. Baseline creatinine was based on the lowest creatinine in the preceding 3 months, or the ideal creatinine based on 75 ml/min/1.73 m^2^ using the MDRD, or the lowest creatinine during ICU admission. AKI severity was based on the highest classification using both serum creatinine and urine output criteria, measured either hourly or averaged over 6 h. AKI incidences were 47%, 38%, and 51% using RIFLE, AKIN, and KDIGO definitions, respectively [20].

The study of Levi et al., compared all three definitions in 190 patients and excluded patients admitted for less than 24 h and patients on RRT before admission. The authors did not specify baseline creatinine and used average urine output over 6 h. AKI incidences were 63% for all three definitions. The distribution of AKI severity differed between AKIN and KDIGO in stages 2 and 3 [21].

Reddy et al., compared AKI incidences using RIFLE and AKIN definitions in 250 patients. All patients with a preexisting renal disease were excluded. Baseline creatinine was based on the lowest serum creatinine during hospital or ICU admission or calculated based on a MDRD of 75 ml/min/1.73 m2. AKI was based on both creatinine and urine output criteria, but measurement of urine output collection was not specified. The authors found higher incidences of AKI using AKIN compared to RIFLE (46% versus 34%) [22].

Wlodzimirow et al., compared incidences in AKI based on RIFLE in 260 ICU patients admitted for at least 48 h and excluded patients with known end stage renal disease. The lowest value of serum creatinine in the preceding 6 months or the estimated serum creatinine based on the MDRD assuming a clearance of 75 ml/min/1.73 m2 was used as reference value. Urine output was measured hourly. Incidences of AKI with and without urine output criteria were 81% and 42%, respectively [15].

Macedo et al., compared incidence in AKI based on AKIN in 75 patients and excluded patients with known end stage renal disease or receiving RRT. The first serum creatinine at ICU admission was used as reference creatinine and urine output was measured hourly. The incidences of AKI according to the AKIN definitions were 28%, 55% and 60% based on serum creatinine, urine output, and a combination, respectively [16].

A multicenter study by Salgado et al., in 627 ICU patients compared AKI incidences using RIFLE and AKIN definitions with and without urine output criteria in patients admitted for at least 48 h. They excluded patients in whom urine output was not possible to quantify, patients with previous urological interventions or patients on RRT. Missing urine output data were estimated using calculating averages. AKI incidences were 69% and 52% using RIFLE and AKIN definitions, with similar AKI incidences when adding urine output criteria (60% with both definitions) [17].

#### AKI incidences in critically ill patients in retrospective studies

Eight studies used a retrospective design to evaluate AKI incidences. No study compared all three definitions. Seven studies compared two definitions and one evaluated the additive value of urine output on AKI incidences. Two studies found AKI incidences of 36% using RIFLE definitions and 26% and 37% using AKIN definitions [23, 24]. Four other studies found AKI incidences in patients with sepsis varying from 56% to 68% depending on the definitions used [25–28]. All studies used serum creatinine and four studies also used urine output criteria. A MDRD of 75 ml/min/1.73 m2 was used as a reference value for serum creatinine in four studies, and in three studies the lowest serum creatinine was used. Urine output was measured as an average over 24 h in one study and not further specified in three studies. One study analyzed the additional value of urine output in AKIN definitions and found that 12% remained unrecognized as having AKI based on serum creatinine [14, 18].

#### AKI incidences in other populations

Ten studies assessed patients that were not critically ill of which four had a prospective and six a retrospective design. Three studies with a prospective and three with a retrospective design compared all three AKI definitions. Incidences varied from 4 to 94%. None of the studies evaluated the additional value of urine output criteria.

## Discussion

We have analyzed differences in AKI incidences based on the three available definitions of RIFLE, AKIN, and KDIGO, each with and without urine output criteria. Urine output criteria increase AKI proportions using all three definitions by adding less sick patients but also enable quicker recognition of AKI by half a day. While RIFLE criteria were initially developed for uniform and standardized reporting of outcomes, our review shows that reported AKI incidences are far from uniform.

The RIFLE AKI definition was developed for lack of clear definitions of AKI and to facilitate uniform reporting renal failure. All AKI definitions, however are validated against mortality because a gold standard for AKI is absent. In fact, AKI criteria are prognostic factors for mortality based on renal symptoms, but the performance as prognostic score for mortality is mediocre [1]. Fulfilling RIFLE creatinine definition requires a higher (at least 50%) creatinine rise than the absolute criterion of 26.4 μmol/l for the AKIN and KDIGO definitions and patients will meet AKIN and KDIGO criteria more easily (Fig. 1 and Additional file 1: Figure S1). Patients who meet RIFLE definition will therefore be sicker as reflected by the APACHE score and have a higher mortality (Table 3). Introduction of a fixed rise in creatinine improves sensitivity but occurs at the cost of specificity.

Adding urine output also leads to a shift in the features of the patients identified to have AKI. A decreased urine output may be the first signal of renal function loss but only 20% will reach the AKI serum creatinine criteria (Fig. 1 and Additional file 1: Table S2). Patients only fulfilling urinary output criteria have a lower APACHE IV score (Table 2). Urine output is frequently not reported in publications for practical reasons. Our data show that AKI incidences will rise considerably when urine output criteria are included while patient outcome probably improves (Table 2) which is corroborated by a recent study [14].

Our overall AKI incidence of 15% when using serum creatinine criteria of the RIFLE definition or 38% when using the combination of serum creatinine and urine output criteria of the AKIN and KDIGO definitions are relatively low compared to the literature.

This could be explained by not having any exclusion criterion in our study, as we included patients with less severe illness. Two third of our patient population was admitted less than 48 h. These design issues could have diluted the AKI incidences. In contrast, we used baseline serum creatinine based on an ideal creatinine using a MDRD of 75 ml/min/1.73 m2 which could have led to overestimation of true incidences as we scored some patients with - previously unknown - CKD as having AKI. The choice which baseline serum creatinine is used has an important effect on incidence of AKI and subsequent on mortality in patients with AKI [29]. However, using an estimated creatinine is widely accepted in studies on AKI for determining baseline creatinine [30]. It is debatable whether these patients have substantial impact on our results as our population contains only 4.2% patients with known CKD. We had only urine output data in 1004 patients which could lead to both over and underestimation of AKI incidences. A possible limitation in our urine output data is the fact that imputation was based on averages using the first known value over the missing hours. Unlike the study of Soliman et al., we did not restrict our backlogging process to 6 h, which could induce possible overestimation of AKI based on the urine output criteria [31].

The considerations mentioned above are applicable to most studies concerning incidence of AKI in critically ill patients. Our presented literature review showed large heterogeneity in patients included, AKI definition used, reference or baseline serum creatinine used, timeframe in which AKI was assessed and whether urine output was used. This inclusion, definition and reference creatinine heterogeneity could explain the differences in AKI incidence in these studies and makes published studies difficult to compare. We intended to conduct a sensitive literature search. However, a sensitive search strategy is always a trade-off between a sufficiently large numbers of hits so that it can be considered sensitive and a sufficiently low numbers of hits so that it can be achievable. With different search criteria other studies might also have been included.

More than a third of patients who only fulfilled AKI criteria based on urine output were admitted after scheduled surgery (Table 2). The significance of oliguria only in the post-operative period is relatively unknown and it is questionable if the prognostic value of AKI diagnosis and classification on oliguria only is applicable to these patients, although patients only fulfilling urine output criteria have higher one year mortality than patients not meeting one of the AKI criteria [14, 32, 33]. The addition of urine output criteria to serum creatinine criteria leads to higher AKI incidences with earlier recognition especially in patients with mild to moderate AKI. It may be possible that more frequent measurements of serum creatinine also reduce the time needed for AKI detection but that would increase costs. Earlier recognition of patients having AKI could provide possibilities for prevention of kidney function deterioration and potentially improve outcomes [34]. Furthermore, especially long-term follow up data are needed to evaluate associations between AKI based on urine output criteria only and kidney function and survival.

## Conclusion

AKIN and KDIGO define a less ill population with a better outcome, compared to RIFLE. Urine output criteria detect patients with AKI 11 h earlier than serum creatinine criteria and gives approximately doubling of AKI incidences in critically ill patients, but most of these patients will never reach creatinine criteria. Consensus definitions have improved research opportunities, however the definitions used, addition of urine output criteria and the patients under study all influence AKI incidences and possibly outcomes.

## Summary

The AKI definition which is used and whether serum creatinine is combined with urine output criteria influences the AKI incidence and the AKI outcome. Adding urine output criteria detects AKI quicker than using only serum creatinine.

## Additional file

Additional file 1: Supplemental material for Incidence, timing and outcome of AKI in critically ill patients varies with the definition used and the addition of urine output criteria. (DOCX 83 kb)

## Abbreviations

AKI: Acute kidney injury
AKIN: Acute Kidney Injury Network
APACHE: Acute Physiology and Chronic Health Evaluation IV
BMI: Body Mass Index
CKD: Chronic kidney disease
GFR: Glomerular Filtration Rate
ICU: Intensive care unit
IQR: Inter quartile range
KDIGO: Kidney Disease Improving Global Outcome
MDRD: Modification of Diet in Renal Disease
METc: Medisch Etische Toetsings commissie (Medical Ethical Testing Committee)
RIFLE: Risk, Injury, Failure
RRT: Renal replacement therapy
