# Supplementary material for: Incidence, timing and outcome of AKI in critically ill patients varies with the definition used and the addition of urine output criteria
Source: BMC Nephrol. 2017 Feb 20;18:70. doi: 10.1186/s12882-017-0487-8 (PMC5319106; doi:10.1186/s12882-017-0487-8)
Supplement: Additional file 1: — Supplemental material for Incidence, timing and outcome of AKI in critically ill patients varies with the definition used and the addition of urine output criteria. (DOCX 83 kb) [file 12882_2017_487_MOESM1_ESM.docx]

Additional file 1

**SUPPLEMENTARY METHODS**

**I. Cohort study**

*Definitions of AKI*

RIFLE definition: RIFLE definition for AKI has five stages of kidney dysfunction. Risk is defined by a rise in serum creatinine of 50% from baseline, a decline in glomerular filtration rate (GFR) of 25% or more compared to baseline or a urine output less than 0.5 ml/kg/h during at least 6 hours. Injury is defined by a doubling of serum creatinine from baseline, a decline in GFR of 50% or more compared to baseline or a urine output less than 0.5 ml/kg/h during at least 12 hours. Failure is defined by an increase of serum creatinine with 300% from baseline, an acute rise in serum creatinine of ≥ 44 μmol/l from baseline, a serum creatinine of ≥ 350 μmol/l, a decline in GFR of 75% compared to baseline, a urine output less than 0.3 ml/kg/h during 24 hours or anuria during at least 12 hours. Loss is defined by persistent loss of kidney function during at least 4 weeks and End Stage Kidney Disease is defined by persistent loss of kidney function during at least 3 months. Loss and End Stage Kidney disease were not used in this study (1)[(1)]. The time span during which the rise in serum creatinine should occur was not mentioned in the original publication by Bellomo, but was defined 7 days by Kellum [(2)] .

AKIN definition: AKIN definition for AKI has three stages of kidney function. Stage 1 is defined by a rise in serum creatinine of at least 26.4 μmol/l, a rise in serum creatinine of 150-200% from baseline or urine output less than 0.5 ml/kg/h for more than 6 hours. Stage 2 is defined by a rise in serum creatinine of 200-300% from baseline or urine output less than 0.5 ml/kg/h during more than 12 hours. Stage 3 is defined by a rise in serum creatinine of more than 300% from baseline, a serum creatinine ≥ 354 μmol/l with an acute increase of at least 44 μmol/l, the need for RRT or a urine output less than 0.3 ml/kg/h during 24 hours or anuria during 12 hours. The rise in serum creatinine has to occur within 48 hours [(3)].

KDIGO definition: KDIGO definition for AKI has three stages of kidney function. Stage 1 is defined by a 1.5-1.9 fold rise in serum creatinine within 7 days compared to baseline, an absolute rise of ≥26.5 μmol/l within 48 hours compared with baseline or a urine output of less than 0.5 ml/kg/h during 6 to 12 hours. Stage 2 is defined by a 2.0-2.9 fold rise in serum creatinine within 7 days compared with baseline or a urine output less than 0.5 ml/kg/h during at least 12 hours. Stage 3 is defined by a 3 fold rise in serum creatinine within 7 days compared with baseline, an absolute serum creatinine ≥354.6 μmol/l, initiation of renal replacement therapy (RRT), a urine output less than 0.3 ml/kg/h during at least 24 hours or anuria during more than 12 hours [(4)]. The different AKI definitions are summarized in supplementary table 1.

**II. Review**

*Search terms*

*RIFLE vs AKIN vs KDIGO*

((AKIN[All Fields] OR ("acute kidney injury"[MeSH Terms] OR ("acute"[All Fields] AND "kidney"[All Fields] AND "injury"[All Fields]) OR "acute kidney injury"[All Fields])))

AND

(("rifle"[All Fields]) OR (("risk"[MeSH Terms] OR "risk"[All Fields]) AND "injury"[All Fields] AND failure[All Fields] AND loss[All Fields] AND end[All Fields] AND stage[All Fields]))

AND

(kdigo[All Fields] OR (("kidney diseases"[MeSH Terms] OR ("kidney"[All Fields] AND "diseases"[All Fields]) OR "kidney diseases"[All Fields] OR ("kidney"[All Fields] AND "disease"[All Fields]) OR "kidney disease"[All Fields]) AND improving[All Fields] AND ("global"[All Fields]) AND outcomes[All Fields]))

*RIFLE vs AKIN*

((AKIN[TiAb] OR ("acute kidney injury"[MeSH Terms] OR ("acute"[TiAb] AND "kidney"[TiAb] AND "injury"[TiAb]) OR "acute kidney injury"[TiAb]))) AND (("rifle"[TiAb]) OR (("risk"[MeSH Terms] OR "risk"[TiAb]) AND "injury"[TiAb] AND failure[TiAb] AND loss[TiAb] AND end[TiAb] AND stage[TiAb])) AND (("critical care"[MeSH Terms] OR ("critical"[TiAb] AND "care"[TiAb]) OR "critical care"[TiAb]) OR ("intensive care"[MeSH Terms] OR ("intensive"[TiAb] AND "care"[TiAb]) OR "intensive care"[TiAb]) OR ICU[TiAb] OR ("critical illness"[MeSH Terms] OR ("critical"[TiAb] AND "illness"[TiAb]) OR "critical illness"[TiAb] OR ("critically"[TiAb] AND "ill"[TiAb]) OR "critically ill"[TiAb]))

*RIFLE vs KDIGO*

(("rifle"[TiAb]) OR (("risk"[MeSH Terms] OR "risk"[TiAb]) AND "injury"[TiAb] AND failure[TiAb] AND loss[TiAb] AND end[TiAb] AND stage[TiAb])) AND (kdigo[TiAb] OR (("kidney diseases"[MeSH Terms] OR ("kidney"[TiAb] AND "diseases"[TiAb]) OR "kidney diseases"[TiAb] OR ("kidney"[TiAb] AND "disease"[TiAb]) OR "kidney disease"[TiAb]) AND improving[TiAb] AND ("global"[TiAb]) AND outcomes[TiAb])) AND (("critical care"[MeSH Terms] OR ("critical"[TiAb] AND "care"[TiAb]) OR "critical care"[TiAb]) OR ("intensive care"[MeSH Terms] OR ("intensive"[TiAb] AND "care"[TiAb]) OR "intensive care"[TiAb]) OR ICU[TiAb] OR ("critical illness"[MeSH Terms] OR ("critical"[TiAb] AND "illness"[TiAb]) OR "critical illness"[TiAb] OR ("critically"[TiAb] AND "ill"[TiAb]) OR "critically ill"[TiAb]))

*AKIN vs KDIGO*

((AKIN[TiAb] OR ("acute kidney injury"[MeSH Terms] OR ("acute"[TiAb] AND "kidney"[TiAb] AND "injury"[TiAb]) OR "acute kidney injury"[TiAb]))) AND (kdigo[TiAb] OR (("kidney diseases"[MeSH Terms] OR ("kidney"[TiAb] AND "diseases"[TiAb]) OR "kidney diseases"[TiAb] OR ("kidney"[TiAb] AND "disease"[TiAb]) OR "kidney disease"[TiAb]) AND improving[TiAb] AND ("global"[TiAb]) AND outcomes[TiAb])) AND (("critical care"[MeSH Terms] OR ("critical"[TiAb] AND "care"[TiAb]) OR "critical care"[TiAb]) OR ("intensive care"[MeSH Terms] OR ("intensive"[TiAb] AND "care"[TiAb]) OR "intensive care"[TiAb]) OR ICU[TiAb] OR ("critical illness"[MeSH Terms] OR ("critical"[TiAb] AND "illness"[TiAb]) OR "critical illness"[TiAb] OR ("critically"[TiAb] AND "ill"[TiAb]) OR "critically ill"[TiAb]))

**SUPPLEMENTARY RESULTS**

Studies resulting from the literature search are listed in table 3.

**SUPPLEMENTARY REFERENCES**

1. Bellomo R, Ronco C, Kellum JA, Mehta RL, Palevsky P, Acute Dialysis Quality Initiative workgroup. Acute renal failure - definition, outcome measures, animal models, fluid therapy and information technology needs: the Second International Consensus Conference of the Acute Dialysis Quality Initiative (ADQI) Group. Crit Care. 2004 Aug;8(4):R204-12.

2. Kellum JA, Levin N, Bouman C, Lameire N. Developing a consensus classification system for acute renal failure. Curr Opin Crit Care. 2002 Dec;8(6):509-14.

3. Mehta RL, Kellum JA, Shah SV, Molitoris BA, Ronco C, Warnock DG, et al. Acute Kidney Injury Network: report of an initiative to improve outcomes in acute kidney injury. Crit Care. 2007;11(2):R31.

4. Kidney Disease: Improving Global Outcomes (KDIGO) Acute Kidney Injury Work Group. KDIGO Clinical Practice Guideline for Acute Kidney Injury. Kidney inter., Suppl. 2012; 2: 1–138. [Internet].; 2012 []. Available from: <http://www.kdigo.org/clinical_practice_guidelines/pdf/KDIGO%20AKI%20Guideline.pdf>.

5. Luo X, Jiang L, Du B, Wen Y, Wang M, Xi X, et al. A comparison of different diagnostic criteria of acute kidney injury in critically ill patients. Crit Care. 2014 Jul 8;18(4):R144.

6. Salgado G, Landa M, Masevicius D, Gianassi S, San-Roman JE, Silva L, et al. Acute renal failure according to the RIFLE and AKIN criteria: a multicenter study. Med Intensiva. 2014 Jun-Jul;38(5):271-7.

7. Reddy NP, Ravi KP, Dhanalakshmi P, Annigeri R, Ramakrishnan N, Venkataraman R. Epidemiology, outcomes and validation of RIFLE and AKIN criteria in acute kidney injury (AKI) in critically ill patients: Indian perspective. Ren Fail. 2014 Jul;36(6):831-7.

8. Levi TM, de Souza SP, de Magalhaes JG, de Carvalho MS, Cunha AL, Dantas JG, et al. Comparison of the RIFLE, AKIN and KDIGO criteria to predict mortality in critically ill patients. Rev Bras Ter Intensiva. 2013 Oct-Dec;25(4):290-6.

9. Wlodzimirow KA, Abu-Hanna A, Slabbekoorn M, Chamuleau RA, Schultz MJ, Bouman CS. A comparison of RIFLE with and without urine output criteria for acute kidney injury in critically ill patients. Crit Care. 2012 Oct 18;16(5):R200.

10. Macedo E, Malhotra R, Claure-Del Granado R, Fedullo P, Mehta RL. Defining urine output criterion for acute kidney injury in critically ill patients. Nephrol Dial Transplant. 2011 Feb;26(2):509-15.

11. Shinjo H, Sato W, Imai E, Kosugi T, Hayashi H, Nishimura K, et al. Comparison of kidney disease: improving global outcomes and acute kidney injury network criteria for assessing patients in intensive care units. Clin Exp Nephrol. 2014 Oct;18(5):737-45.

12. Ratanarat R, Skulratanasak P, Tangkawattanakul N, Hantaweepant C. Clinical accuracy of RIFLE and Acute Kidney Injury Network (AKIN) criteria for predicting hospital mortality in critically ill patients with multi-organ dysfunction syndrome. J Med Assoc Thai. 2013 Feb;96 Suppl 2:S224-31.

13. Kim WY, Huh JW, Lim CM, Koh Y, Hong SB. A comparison of acute kidney injury classifications in patients with severe sepsis and septic shock. Am J Med Sci. 2012 Nov;344(5):350-6.

14. Han SS, Kang KJ, Kwon SJ, Wang SJ, Shin SH, Oh SW, et al. Additional role of urine output criterion in defining acute kidney injury. Nephrol Dial Transplant. 2012 Jan;27(1):161-5.

15. Chang CH, Lin CY, Tian YC, Jenq CC, Chang MY, Chen YC, et al. Acute kidney injury classification: comparison of AKIN and RIFLE criteria. Shock. 2010 Mar;33(3):247-52.

16. Joannidis M, Metnitz B, Bauer P, Schusterschitz N, Moreno R, Druml W, et al. Acute kidney injury in critically ill patients classified by AKIN versus RIFLE using the SAPS 3 database. Intensive Care Med. 2009 Oct;35(10):1692-702.

17. Lopes JA, Fernandes P, Jorge S, Goncalves S, Alvarez A, Costa e Silva Z, et al. Acute kidney injury in intensive care unit patients: a comparison between the RIFLE and the Acute Kidney Injury Network classifications. Crit Care. 2008;12(4):R110.

18. Bagshaw SM, George C, Bellomo R, ANZICS Database Management Committe. A comparison of the RIFLE and AKIN criteria for acute kidney injury in critically ill patients. Nephrol Dial Transplant. 2008 May;23(5):1569-74.

19. Neves M, Fidalgo P, Goncalves C, Leitao S, Santos RM, Carvalho A, et al. Acute kidney injury in an internal medicine ward in a Portuguese quaternary hospital. Eur J Intern Med. 2014 Feb;25(2):169-72.

20. Rodrigues FB, Bruetto RG, Torres US, Otaviano AP, Zanetta DM, Burdmann EA. Incidence and mortality of acute kidney injury after myocardial infarction: a comparison between KDIGO and RIFLE criteria. PLoS One. 2013 Jul 23;8(7):e69998.

21. Roy AK, Mc Gorrian C, Treacy C, Kavanaugh E, Brennan A, Mahon NG, et al. A Comparison of Traditional and Novel Definitions (RIFLE, AKIN, and KDIGO) of Acute Kidney Injury for the Prediction of Outcomes in Acute Decompensated Heart Failure. Cardiorenal Med. 2013 Apr;3(1):26-37.

22. Sampaio MC, Maximo CA, Montenegro CM, Mota DM, Fernandes TR, Bianco AC, et al. Comparison of diagnostic criteria for acute kidney injury in cardiac surgery. Arq Bras Cardiol. 2013 Jul;101(1):18-25.

23. Karapanagiotou A, Dimitriadis C, Papadopoulos S, Kydona C, Kefsenidis S, Papanikolaou V, et al. Comparison of RIFLE and AKIN criteria in the evaluation of the frequency of acute kidney injury in post-liver transplantation patients. Transplant Proc. 2014 Nov;46(9):3222-7.

24. Fujii T, Uchino S, Takinami M, Bellomo R. Validation of the Kidney Disease Improving Global Outcomes Criteria for AKI and Comparison of Three Criteria in Hospitalized Patients. Clin J Am Soc Nephrol. 2014 May;9(5):848-54.

25. Li Z, Cai L, Liang X, Du Z, Chen Y, An S, et al. Identification and Predicting Short-Term Prognosis of Early Cardiorenal Syndrome Type 1: KDIGO Is Superior to RIFLE or AKIN. PLoS One. 2014 Dec 26;9(12):e114369.

26. Shacham Y, Leshem-Rubinow E, Ziv-Baran T, Gal-Oz A, Steinvil A, Ben Assa E, et al. Incidence and mortality of acute kidney injury in acute myocardial infarction patients: a comparison between AKIN and RIFLE criteria. Int Urol Nephrol. 2014 Dec;46(12):2371-7.

27. Bastin AJ, Ostermann M, Slack AJ, Diller GP, Finney SJ, Evans TW. Acute kidney injury after cardiac surgery according to Risk/Injury/Failure/Loss/End-stage, Acute Kidney Injury Network, and Kidney Disease: Improving Global Outcomes classifications. J Crit Care. 2013 Aug;28(4):389-96.

28. Han D, Liu Z, Han Q, Li Z, Zhang G, Qiu J, et al. Acute kidney injury in patients with hemorrhagic fever with renal syndrome caused by Hantaan virus: comparative evaluation by RIFLE and AKIN criteria. Vector Borne Zoonotic Dis. 2011 Jun;11(6):723-30.

**Supplementary table 1**.

|  | **RIFLE** | **AKIN** | **KDIGO** | **RIFLE, AKIN & KDIGO** |
| --- | --- | --- | --- | --- |
| **Serum creatinine** |  |  |  | **Urine output** |
| **1 or Risk** | creatinine *1.5  Or  GFR decrease >25% | creatinine + ≥ 26.4 µmol/l  Or  creatinine 150-200% (*1.5-2.0) | creatinine *1.5-1.9  Or  creatinine + ≥ 0.3 mg/dl (26.5 µmol/l) | <0.5 ml/kg/h ≥ 6u |
| **2 or Injury** | creatinine *2  Or  GFR decrease >50% | creatinine 200-300% (*>2.0-3.0) | creatinine * 2.0-2.9 | <0.5 ml/kg/h ≥ 12u |
| **3 or Failure** | creatinine *3  Or  creatinine ≥ 4 mg/dl (350 µmol/l) with acute increase ≥ 0.5 (44 µmol/l) mg/dl  Or  GFR decrease 75% | creatinine >300% (*>3.0)  Or  creatinine ≥ 4 mg/dl (354 µmol/l) with acute increase ≥ 0.5 mg/dl (44 µmol/l)  Or  RRT | creatinine *3  Or  creatinine ≥ 4 mg/dl (354 µmol/l)  Or  RRT | < 0.3 ml/kg/h ≥ 24u  Or  Anuria ≥ 12u |
| **Time interval** | < 1-7 days | < 48 hours | < 7 days |  |

**Supplementary table 2.**

|  |  | **AKI at admission** | | | | |  | **No AKI at admission** | | | | | | **All patient**s |
| --- | --- | --- | --- | --- | --- | --- | --- | --- | --- | --- | --- | --- | --- | --- |
|  |  | ***UO criteria*** | | | |  |  | ***UO criteria*** | | | | |  |  |
|  |  | R/1 | I/2 | F/3 | Missing | Total |  | R/1 | I/2 | F/3 | No AKI | Missing | Total |  |
| **SCr criteria** | **RIFLE** |  |  |  |  |  |  |  |  |  |  |  |  |  |
|  | Risk | 3 | 7 | 1 | 14 | 25 |  | 7 | 14 | 4 | 17 |  | 42 |  |
|  | Injury | 5 | 8 | 1 | 28 | 42 |  | 3 | 6 | 7 | 4 |  | 20 |  |
|  | Failure | 10 | 20 | 18 | 27 | 75 |  | 1 | 2 | 2 | 1 |  | 6 |  |
|  | No AKI | 0 | 0 | 0 | 0 | 0 |  | 170 | 88 | 7 | 559 | 335 | 1159 |  |
|  | Total | 18 | 35 | 20 | 69 | 142 |  | 181 | 110 | 20 | 581 | 335 | 1227 | 1369 |
|  |  |  |  |  |  |  |  |  |  |  |  |  |  |  |
| **SCr criteria** | **AKIN** |  |  |  |  |  |  |  |  |  |  |  |  |  |
|  | 1 | 8 | 15 | 0 | 52 | 75 |  | 18 | 18 | 1 | 25 |  | 62 |  |
|  | 2 | 5 | 9 | 2 | 25 | 41 |  | 5 | 7 | 6 | 1 |  | 19 |  |
|  | 3 | 10 | 20 | 33 | 21 | 84 |  | 0 | 3 | 6 | 4 |  | 13 |  |
|  | No AKI |  |  |  |  |  |  | 153 | 73 | 4 | 531 | 314 | 1075 |  |
|  | Total | 23 | 44 | 23 | 110 | 200 |  | 176 | 101 | 17 | 561 | 314 | 1169 | 1369 |
|  |  |  |  |  |  |  |  |  |  |  |  |  |  |  |
| **SCr criteria** | **KDIGO** |  |  |  |  |  |  |  |  |  |  |  |  |  |
|  | 1 | 8 | 15 | 0 | 49 | 72 |  | 14 | 19 | 2 | 20 |  | 55 |  |
|  | 2 | 5 | 6 | 1 | 26 | 38 |  | 3 | 5 | 3 | 1 |  | 12 |  |
|  | 3 | 10 | 23 | 22 | 33 | 88 |  | 1 | 3 | 8 | 4 |  | 16 |  |
|  | No AKI |  |  |  |  |  |  | 158 | 74 | 4 | 535 | 317 | 1088 |  |
|  | Total | 23 | 44 | 23 | 108 | 198 |  | 176 | 101 | 17 | 560 | 317 | 1171 | 1369 |

**Supplementary table 3.**

| Study | N | Baseline creatinine | UO criteria and method | Exclusion criteria | Setting | RIFLE | AKIN | KDIGO |
| --- | --- | --- | --- | --- | --- | --- | --- | --- |
| Prospective studies in ICU patients | | | | | | | | |
| **Luo (2014)** [(5)] | 3107 | Lowest SCr in the past 3 months  If unknown:  RIFLE and KDIGO: estimated (MDRD (75 ml/min/1.73m2)) (N=120) baseline or lowest SCr during ICU admission (N=634)  AKIN: SCr at ICU admission | Hourly or total volume in a 6 hour period | ESKD  RRT  Kidney transplantation <3 months  ICU admission <24 hours | ICU | Total 46.9% | Total 38.4% | Total 51% |
| **Salgado (2014)** [(6)] | 627 | MDRD (75 ml/min/1.73m2) | Average hourly UO based on two-hourly collection by CAD or based on micturition episodes. | Inability to quantify UO at any time during study period  Surgical instrumentation of the urinary tract  RRT (new onset or chronic)  ICU admission <48 hours | ICU | Total: 69,4%   - urine criteria: 59,5% - creatinine criteria: 34,7% | Total: 51,8%   - urine criteria: 59,5% - creatinine criteria: 25,3% |  |
| **Reddy (2014)** [(7)] | 250 | Lowest of MDRD (75 ml/min/1.73m2), during hospital admission or ICU admission | Not stated but used for classification | CCU admission  Any known pre-existing renal disease (baseline SCr >1.3 mg/dl men, >1.2 mg/dl women or by decision of consulting nephrologist) | ICU | Risk: 25.3%  Injury: 27.7%  Failure: 46.9%  Total:34.3% | 1: 44.1%  2: 20.7%  3:35.2%  Total: 45.9% |  |
| **Levi (2013)** [(8)] | 190 | Not stated | UO measured every 6 hours | ICU admission <24 hours  RRT before admission | ICU | Risk: 30.5%  Injury: 14.7%  Failure 17.4%  Total 62.6% | 1: 37.4%  2: 7.9%  3: 17.9%  Total 63.2% | 1: 37.4%  2: 7.4%  3: 18.4%  Total 63.2% |
| **Wlodzimirow (2012)** [(9)] | 260 | Lowest of SCr <6 months of ICU admission or MDRD (75 ml/min/1.73m2) | UO measured hourly | ICU admission < 48 hours  Known ESKD  RRT before admission | ICU | Risk: 18%  Injury: 19%  Failure: 3%  Total with UO 81%  Total without 42% |  |  |
| **Macedo (2011)** [(10)] | 75 | First measured SCr at ICU admission | UO measured by a digital continuous urine meter | Known ESKD  RRT before admission | ICU |  | Total:   - Creatinine 4/75 - UO 24/75 - Creatinine and/or UO 17/75 |  |
| Retrospective studies in ICU patients | | | | | | | | |
| **Shinjo (2014)** [(11)] | 2579 | Lowest SCr during hospitalisation | Not used | Known ESKD  RRT before admission  Kidney transplant  ICU admission < 24 hours | ICU |  | 1: 20.7%  2: 3.4%  3: 5.4%  Total 29.5% | 1: 24.8%  2: 6.2%  3: 7.5%  Total 38.4% |
| **Ratanarat (2013)** [(12)] | 300 | Not stated (abstract only) | Not stated (abstract only) | Not stated (abstract only) | ICU | Risk: 12.7%  Injury: 20.7%  Failure: 33.3%  Total 66.7% | 1: 16%  2: 13.3%  3: 47.3%  Total 76.7% |  |
| **Kim (2012)** [(13)] | 326 | Lowest SCr <3 months or MDRD (75 ml/min/1.73m2) if unknown (N=61)  AKIN: SCr at ICU admission | UO measured hourly | (chronic) RRT before admission  ICU admission <24 hours | ICU patients with severe sepsis/septic shock | Risk (48h): 21,0%  Risk (72h): 13,6%  Injury (48h): 21.6%  Injury (72h): 20.8%  Failure (48h): 20.3%  Failure (72h): 21.1%  Total (48h): 62.9%  Total (72h): 55.5% | 1 (48h): 22,8%  1 (72h): 17,0%  2 (48h): 17.9%  2 (72h): 15.1%  3 (48h): 25.1%  3 (72h): 27.2%  Total (48h): 65.6%  Total (72h): 59.2% |  |
| **Han (2012)** [(14)] | 1625 | Lowest SCr <7 days | UO measured hourly | Known CKD  Known ESKD | ICU |  | 1:   - Creatinine: 548/1625   UO: 215/1625  2:   - Creatinine: 100/1625   UO: 113/1625  3:   - Creatinine: 179/1625   UO: 90/1625  Total: 57% ((1625-699)/1625) |  |
| **Chang (2010)** [(15)] | 291 | First SCr during hospitalization or MDRD (75 ml/min/1.73m2) if admitted directly to the ICU (N=20) | Not stated / used | Chronic uremic patients on RRT  Hospital admission <24 hours | ICU patients with septic shock, ARDS or hepatic cirrhosis | Risk: 13.1%  Injury: 17.9%  Failure: 29.9%  Total: 60.8% | 1: 19.6%  2: 16.8%  3: 12.3%  Total: 68% |  |
| **Joannidis (2009)** [(16)] | 16.784 | MDRD (75 ml/min/1.73m2) | Average UO measured over 24h | Chronic renal failure according to SAPS 3 handbook (a.o. RRT)  Kidney transplantation | ICU | Risk: 7.6%  Injury: 11.1%  Failure: 16.8%  Total: 35.5% | 1: 7.5%  2: 7.2%  3: 13.8%  Total: 28.5% |  |
| **Lopes (2008)** [(17)] | 662 | MDRD (75 ml/min/1.73m2) | UO measured hourly | Chronic kidney disease  RRT before admission | ICU | Risk: 14.7%  Injury: 11%  Failure: 18.1%  Total: 43.8% | 1: 21.1%  2: 10.1%  3: 19.2%  Total: 50.4% |  |
| **Bagshaw (2008)** [(18)] | 120.123 | MDRD (75 ml/min/1.73m2) | Average UO measured over 24h (patient weight assumed 70kg) | ESKD  RRT before admission  Kidney transplantation | ICU | Risk: 16.2%  Injury: 13.6%  Failure: 6.3%  Total: 36.1% | 1: 18.1%  2: 10.1%  3: 8.9  Total: 37.1% |  |
| Prospective studies in non−ICU patients | | | | | | | | |
| **Neves (2014)** [(19)] | 69 | Not stated | Not stated | ICU admission < 72h  Stage 5 CKD  Renal transplantation | Patients 72h in the internal ward with AKI or exacerbation of CKD on admission or developed during hospitalisation | Risk: 26.1%  Injury: 23.2%  Failure: 45%  Total: 94.2% | 1: 29.0%  2: 26.1%  3: 29%  Total: 84.1% | 1: 29.0%  2: 26.1%  3: 29%  Total: 84.1% |
| **Rodrigues (2013)** [(20)] | 1050 | MDRD (75 ml/min/1.73m2)? | Not used | Hospital admission < 48h | (N)STEMI | Risk: 9.6%  Total: 14.8% |  | 1: 30.8%  Total: 36.6% |
| **Roy (2013)** [(21)] | 637 | SCr < 1year of admission | Not used | RRT before admission  Renal transplantation | Patients admitted with systolic heart failure | Total: 25.6% | Total: 27.9% | Total: 26.5% |
| **Sampaio (2013)** [(22)] | 321 | SCr pre-operative | Not used | SCr >= 2.5 mg/dl pre-operative | Patients admitted for myocardial revascularization surgery, valve replacement or both | Total: 15% | Total: 51% | Total: 19% |
| Retrospective studies in non−ICU patients | | | | | | | | |
| **Karapanagiotou (2014)** [(23)] | 71 | Not stated | UO measured hourly | Liver retransplantation  Liver- & kidney transplantation  Acute hepatic failure | Liver transplant | Risk: 12,7%  Injury: 12,7%  Failure 14,1%  Total: 39,4% | 1: 22.5%  2: 7%  3: 22.5%  Total: 52.1% |  |
| **Fujii (2014)** [(24)] | 49.518 | Most recent SCr 1-12 months before admission or based on MDRD (75 ml/min/1.73m2) | Not used | CKD5 at admission and RRT  RRT before admission  Hospital admission <2 days | Hospitalized patients | Total: 16.1% | Total: 16.6% | 1: 70.9%  2: 17.1%  3: 12.0%  Total: 18.3% |
| **Li (2014)** [(25)] | 1005 | Lowest SCr at admission (if in normal range) or SCr < 3 months of admission (27%).  MDRD (75 ml/min/1.73m2) if no SCr was available | Not used | No SCr available in first 7 days of hospitalization  Hospitalisation <48 hours  ESRD with RRT  SCr at admission > = 3.5 mg/dl  Malignant tumor  Cardiac surgery  Contrast medium associated AKI | Hospitalized patients with acute heart failure | Risk: 14.0%  Injury: 11.5%  Failure: 6.6%  Total: 32.1% | 1: 15.6%  2: 10.0%  3: 9.1%  Total: 34.7% | 1: 19.3%  2: 10.3%  3: 9.3%  Total: 38.9% |
| **Shacham (2014)** [(26)] | 1033 | SCr at hospital admission | Not used | Admission <24 hours  RRT before admission | STEMI | Risk: 1,9%  Total: 3,9% | 1: 7,6%  Total: 9,6% |  |
| **Bastin (2013)** [(27)] | 1922 | Most recent SCr (pre−admission clinic or hospital admission) | Not used | VAD  ECMO  Cardiac transplantation  ≥ 2 CPB episodes in 1 admission  RRT before surgery  Death <24 hours after surgery | Patients after cardiac surgery with CPB | Risk: 17.9%  Injury: 5.2%  Failure: 1.9%  Total: 24.9% | 1: 16.9%  2: 1.8%  3: 7.2%  Total: 25.9% | Same as AKIN |
| **Han (2011)** [(28)] | 120 | SCr at hospital admission | Average UO over 24 hours (patient weight assumed 70kg) | CKD  RRT before admission  Hypertension  DM  Admission <24 hours | Hantaanvirus | Risk (48h): 14.2%  Risk (1w): 7.5%  Injury (48h): 10.8%  Injury (1w): 13.3%  Failure (48h): 54.2%  Failure (1w): 63.3%  Total (48h): 79.2%  Total (1w): 84.2% | 1 (48h): 17.5%  1 (1w): 12.5%  2 (48h): 10%  2 (1w): 13.3%  3 (48h): 55.0%  3 (1w): 63.3%  Total (48h): 82.5%  Total (1w): 89.2% |  |

**Supplementary figure 1.**


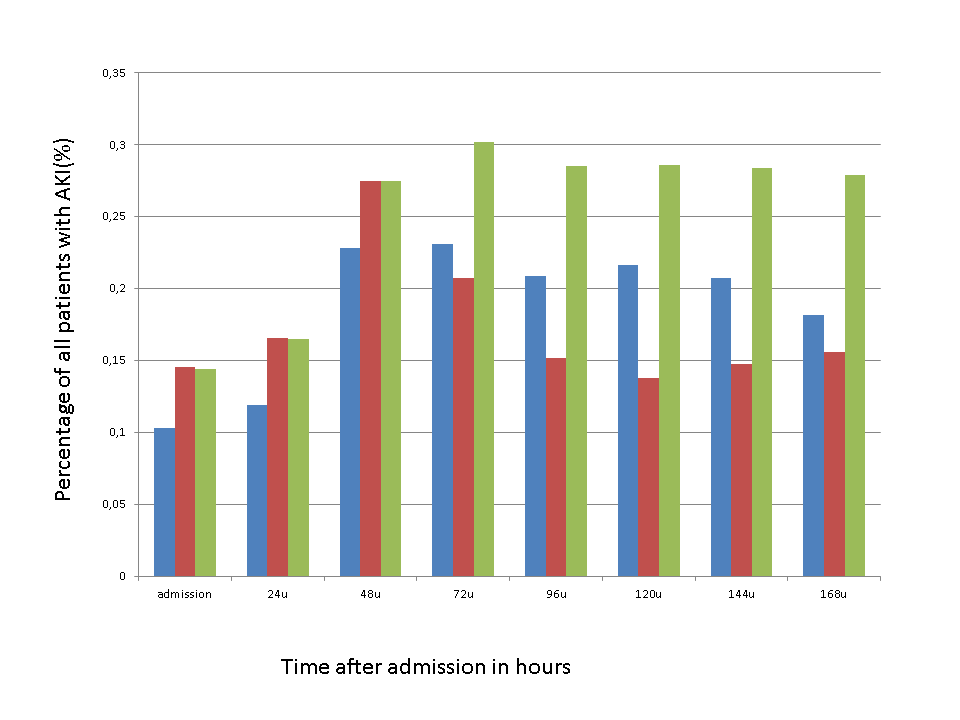


**SUPPLEMENTARY LEGENDS**

**Supplementary table 1. RIFLE, AKIN and KDIGO defintions with serum creatinine and urine output criteria.**

RIFLE: ‘risk’, ‘injury’, ‘failure’, ‘loss’ and ‘end-stage’ definition.

AKIN: acute kidney injury network definition

KDIGO: kidney disease improving global outcome definition.

GFR: glomerular filtration rate

UO: urine output

**Supplementary table 2. Severity of AKI during first week grouped by patients with AKI at admission and those who developed AKI during the first week of ICU admission by RIFLE, AKIN and KDIGO definition based on serum creatinine and urine output criteria.**

Left: patients with AKI at admission. Right: patients without AKI at admission. Numbers of patients with AKI according to serum creatinine and urine output grouped by severity and AKI definition.

**Supplementary table 3. Studies used in the review.**

**Supplementary figure 1. Incidences of AKI during the first week after admission according to the serum creatinine criteria of the RIFLE, AKIN and KDIGO definitions.**

y-axis: proportions of all patients

x-axis: time (in hours) following admission to the Intensive Care Unit (ICU).

Blue columns: proportion according to RIFLE definition, red columns: proportions according to AKIN definition, green columns: proportions according to KDIGO definition.
